# Second Primary Subglottic Laryngeal Carcinoma After Cyclophosphamide, Doxorubicin, Vincristine, and Prednisolone (CHOP) Chemotherapy for Non-Hodgkin Lymphoma

**DOI:** 10.7759/cureus.101528

**Published:** 2026-01-14

**Authors:** Cijo Sanal, Isha Jaiswal, Pratikshya Biswal, Lahunshisha Kharbuli

**Affiliations:** 1 Department of Radiotherapy, Institute of Medical Sciences, Banaras Hindu University, Varanasi, IND

**Keywords:** chop chemotherapy, diffuse large b-cell lymphoma, non-hodgkin lymphoma, second primary malignancy, subglottic carcinoma, therapy related cancer

## Abstract

Second primary malignancies are a recognised late complication in lymphoma survivors, particularly in those treated with alkylating agents and topoisomerase II inhibitors. While therapy-related haematological malignancies are well documented, solid tumours of the head and neck remain uncommon.

We report the case of a 72-year-old man with a history of stage IV high-grade non-Hodgkin lymphoma, most likely diffuse large B-cell lymphoma (DLBCL) based on morphology, treated with eight cycles of cyclophosphamide, doxorubicin, vincristine, and prednisolone (CHOP) chemotherapy in 2018. He achieved remission but returned five years later with progressive hoarseness and stridor. Emergency tracheostomy was performed for airway stabilisation. Contrast-enhanced CT revealed an enhancing subglottic lesion with extension into the glottis, and biopsy confirmed moderately differentiated squamous cell carcinoma. The absence of conventional risk factors such as tobacco or alcohol use favoured a therapy-related pathogenesis.

To our knowledge, this is the first reported case of subglottic squamous cell carcinoma arising after CHOP chemotherapy for non-Hodgkin lymphoma in the absence of conventional risk factors. This underscores the importance of lifelong vigilance in lymphoma survivors and the prompt evaluation of new airway symptoms to enable timely diagnosis and potentially curative treatment.

## Introduction

Second primary malignancies (SPMs) are seen in patients exposed to alkylating agents and topoisomerase II inhibitors in lymphoma patients [1,2]. While therapy-related leukaemias are well documented, solid tumours, especially in the head and neck region, are less frequently reported. Cyclophosphamide, doxorubicin, vincristine, and prednisolone (CHOP) chemotherapy remains the historical backbone of treatment for diffuse large B-cell lymphoma (DLBCL) [3]. The addition of rituximab has become standard in most settings [4]. It includes agents with known carcinogenic potential, and long-term survivors may remain at risk of therapy-related malignancies.

This patient, treated with CHOP chemotherapy for stage IV non-Hodgkin lymphoma, developed a second primary squamous cell carcinoma (SCC) of the subglottic larynx with glottic extension five years after achieving remission, despite having no conventional head and neck cancer risk factors such as tobacco or alcohol use. This rare presentation underscores the importance of long-term vigilance, early evaluation of new airway symptoms, and lifelong surveillance in lymphoma survivors.

## Case presentation

A 72-year-old man, with no known comorbidities and no lifetime history of smoking, smokeless tobacco use, alcohol consumption, or second-hand smoke exposure, first presented in 2018 with a gradually enlarging left cervical swelling and mild dysphagia. He was a retired blood bank employee with no occupational exposure to industrial toxins, chemicals, or pollutants and no history of professional voice abuse. There was no history suggestive of chronic laryngopharyngeal reflux, and no prior exposure to head and neck irradiation. On examination, multiple firm, non-tender left cervical lymph nodes were palpable, with no overlying skin changes.

**Figure 1:**
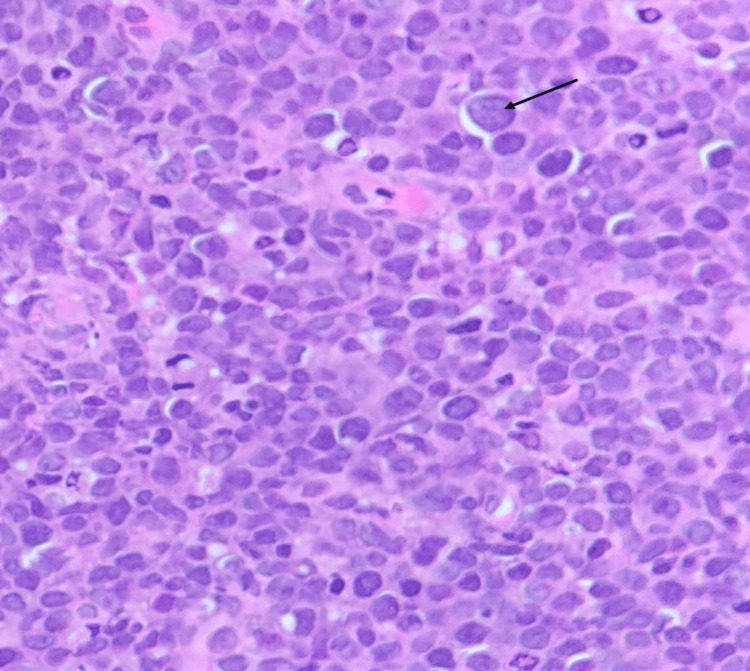
Biopsy from a cervical lymph node Biopsy from a cervical lymph node showing sheets of large atypical lymphoid cells with prominent nucleoli (arrow), (haematoxylin and eosin (H&E) stain, ×40 magnification, scale bar = 100 μm).

**Figure 2:**
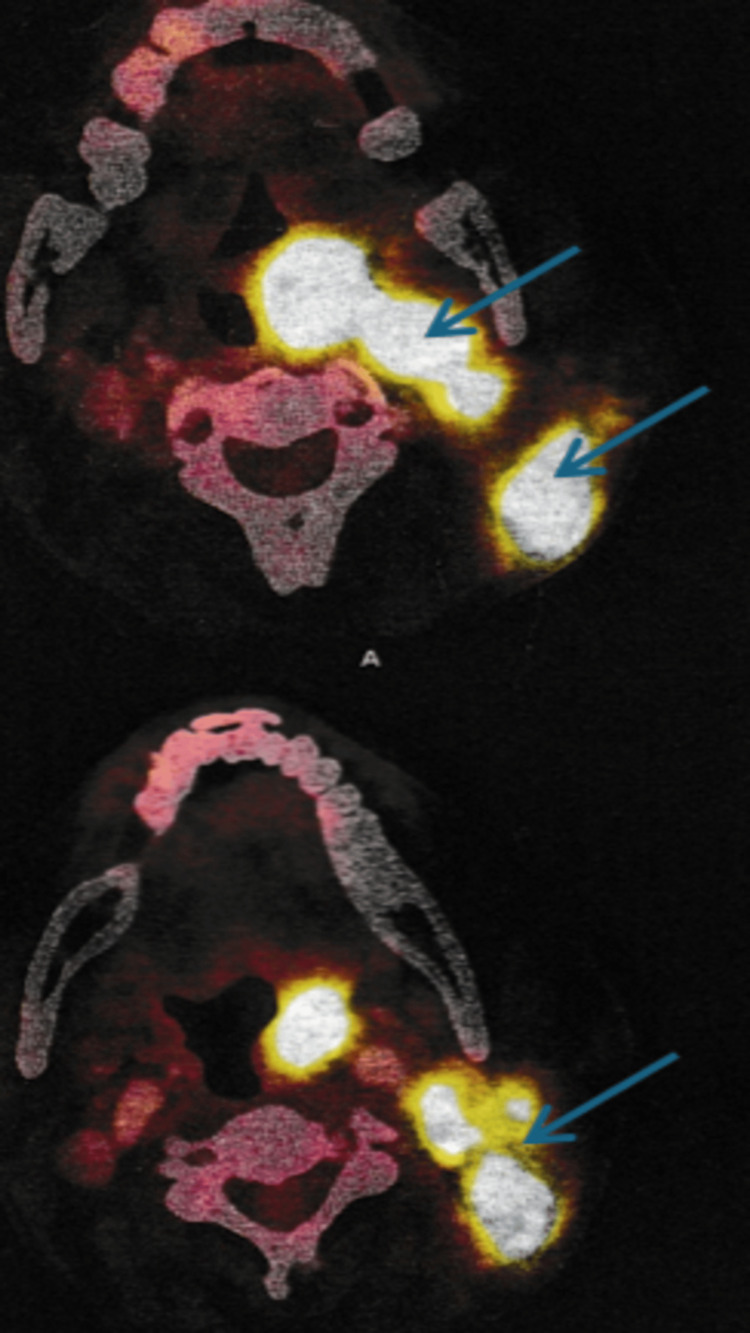
Fused PET-CT images of the neck Fused PET-CT images of the neck showing FDG uptake in right cervical lymph nodes (arrows), consistent with metabolically active nodal disease. Axial fused PET-CT images demonstrate multiple hypermetabolic nodes with SUVmax of 19.

**Figure 3:**
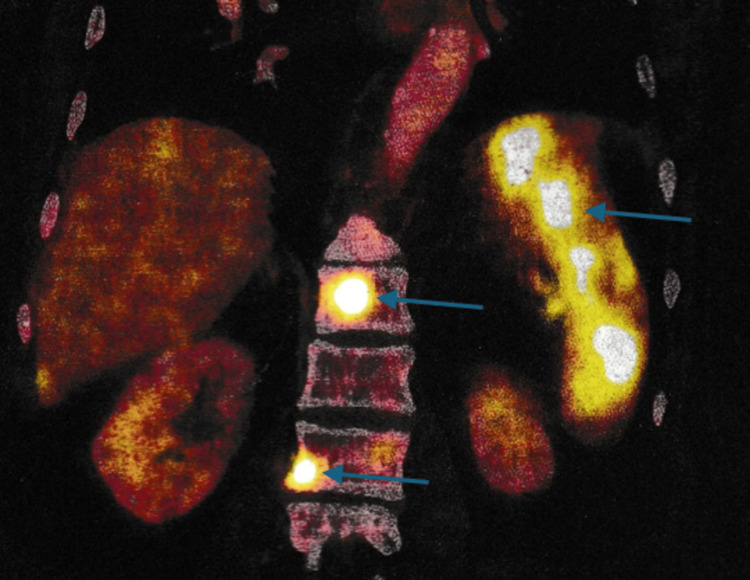
Whole-body PET-CT (coronal fused image) Whole-body PET-CT (coronal fused image) demonstrating widespread hypermetabolic disease. Intense fluorodeoxyglucose (FDG) uptake is noted in the spleen and multiple vertebral bodies (arrows), consistent with disseminated lymphoma.

A biopsy from the oropharyngeal region revealed ulceration, necrosis, and clusters of large atypical lymphoid cells with prominent nucleoli, raising a strong suspicion for high-grade non-Hodgkin lymphoma (Figure 1). The morphology was most consistent with DLBCL; however, immunohistochemistry, which is required for definitive subtyping, was not performed due to financial constraints of the patient. Whole-body PET-CT demonstrated widespread hypermetabolic disease involving cervical and supraclavicular nodes (largest ~3 cm), the pharyngeal wall, nasopharynx, base of tongue, tonsillar pillars, liver, spleen, and bones (ribs, spine, pelvis, shoulder) (Figures 2-3). No abnormal uptake was seen in the larynx or thyroid. These findings were consistent with advanced-stage (stage IV) lymphoma.

The case was reviewed in a multidisciplinary tumour board, and treatment was initiated with CHOP chemotherapy (cyclophosphamide 750 mg/m² IV day 1, doxorubicin 50 mg/m² IV day 1, vincristine 1.4 mg/m² IV day 1 with a maximum dose of 2 mg, and prednisolone 100 mg orally days 1-5), repeated every three weeks. Rituximab was not administered due to financial constraints. Based on his age (>60 years), advanced stage, elevated LDH (509 U/L), ECOG performance status of 1, and multiple extra nodal sites (liver, spleen, bone, pharyngeal wall), his International Prognostic Index (IPI) score was 4/5, placing him in the high-risk group. In view of this risk profile and omission of rituximab, eight cycles were administered. The regimen was well tolerated with no significant toxicities or delays, and no radiotherapy was given. He achieved clinical remission and remained disease-free for two years, after which he defaulted from follow-up.

**Figure 4:**
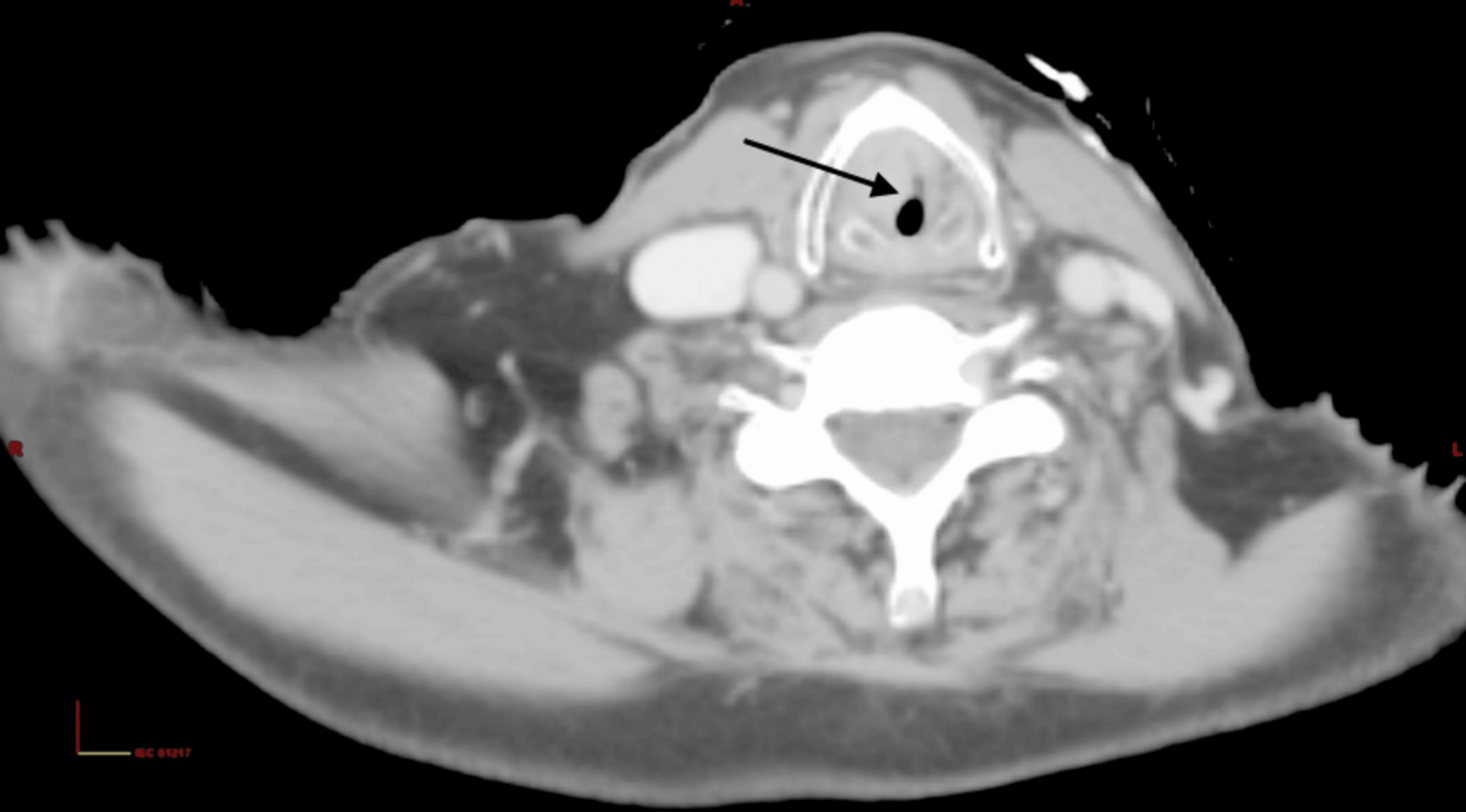
Contrast-enhanced CT (CECT) of the neck and thorax (axial view) Contrast-enhanced CT (CECT) of the neck and thorax (axial view) showing an ill-defined, mildly enhancing soft-tissue lesion in the right subglottic larynx (arrow).

**Figure 5:**
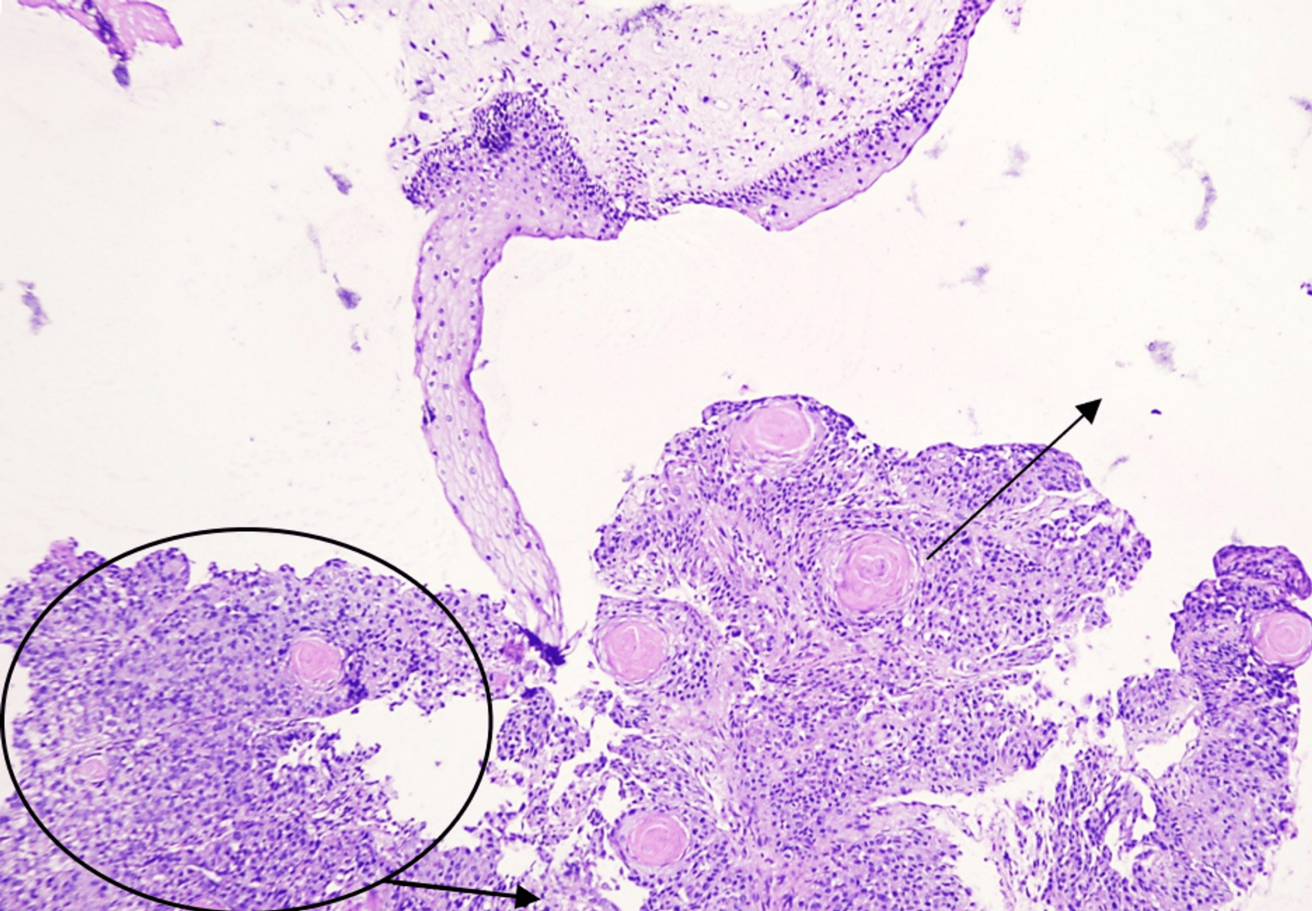
Biopsy from the subglottic larynx Biopsy from the subglottic larynx showing squamous epithelium with tumor cells disposed in sheets (circled) and formation of keratin pearls (arrow). Stained with haematoxylin and eosin (H&E), viewed under ×100 magnification, scale bar = 50 μm.

Five years later, the patient presented with progressive hoarseness, noisy breathing, and stridor. On arrival, he was in respiratory distress and underwent emergency tracheostomy. Contrast-enhanced CT of the neck and thorax revealed an ill-defined, mildly enhancing soft-tissue lesion in the right subglottic larynx (11.8 × 12.0 × 18.7 mm), extending superiorly to the right true vocal cord, causing critical airway narrowing but without cartilage invasion or lymphadenopathy (Figure 4). Direct laryngoscopy demonstrated an irregular exophytic mass on the right true vocal cord with reduced mobility and fixation; the left cord was not visualised, but bilateral arytenoids, pyriform sinuses, and tongue base were normal (no intraoperative image was available for inclusion). Biopsy confirmed moderately differentiated SCC (Figure 5).

Given the absence of nodal disease, systemic relapse, or B symptoms, the lesion was staged as T3N0 subglottic carcinoma with glottic extension, consistent with a SPM rather than lymphoma recurrence. The case was discussed in a multidisciplinary tumour board, and definitive concurrent chemoradiotherapy using linear accelerator with 6-MV beam energy was planned.

## Discussion

SPMs are a well-recognised late complication in lymphoma survivors, particularly among those exposed to alkylating agents and topoisomerase II inhibitors [1,2]. While therapy-related haematological malignancies are the most frequently reported, solid tumours are rare.

Our patient received CHOP chemotherapy alone, without rituximab, due to financial constraints, while R-CHOP is the recommended standard in most settings [4]. This therapeutic exposure, combined with the absence of conventional risk factors such as smoking or alcohol use, supports a therapy-related pathogenesis for the subglottic SCC that developed five years after lymphoma remission.

Our patient developed a SCC of the subglottic larynx with glottic extension five years after completing eight cycles of CHOP chemotherapy for stage IV DLBCL. He had no conventional risk factors for head and neck SCC, including tobacco or alcohol use, occupational or environmental toxin exposure, professional voice abuse, or symptoms of chronic laryngopharyngeal reflux, and the larynx was uninvolved at the time of lymphoma diagnosis. Taken together, these findings raise the possibility of a therapy-associated SPM rather than a field cancerization effect, although a definitive causal relationship cannot be established.

To date, very few reports describe upper aerodigestive tract SCC following CHOP. Tangsirapat et al. [5] reported a hypopharyngeal/laryngeal carcinoma with subglottic extension occurring 18 months after CHOP for testicular lymphoma in a smoking patient, where tobacco may have contributed to carcinogenesis. Li et al. [6] described two DLBCL patients who developed human papillomavirus (HPV)-16-positive tongue-base SCC within three years of R-CHOP. One achieved long-term remission after chemoradiotherapy, while the other died within 27 months, underscoring the aggressive nature of these tumours. Together with our case, these reports highlight that HNSCC may emerge metachronously in lymphoma survivors, even without traditional risk factors, and mandate prolonged surveillance.

Evidence from larger cohorts supports this association. Travis et al. [1] in a study of 6,171 NHL survivors, demonstrated a dose-response relationship between cumulative cyclophosphamide exposure and bladder cancer risk, highlighting the potent mutagenic potential of this agent beyond the urinary tract. Similarly, Geurts et al. [2] found a 1.5-fold increased risk of secondary solid tumours among DLBCL survivors treated with higher cumulative doses of cyclophosphamide (>4500 mg/m²) and doxorubicin (>300 mg/m²), with the upper aerodigestive tract among the commonly affected sites. Other reports, such as Wei et al. [7], reported a metastatic leiomyosarcoma developing seven years after CHOP, illustrating that diverse carcinomas and sarcomas may emerge long after lymphoma remission.

Registry data confirm these risks at the population level. Parsons et al. [8] demonstrated that chemotherapy-treated lymphoma survivors have a significantly higher risk of SPMs compared with both non-treated patients and the general population. Given our patient’s cumulative exposure (>4500 mg/m² cyclophosphamide and >300 mg/m² doxorubicin), he falls into a high-risk category for therapy-related malignancies.

This case, therefore, reinforces the need for lifelong clinical vigilance in lymphoma survivors, particularly for new airway or voice symptoms. Early recognition of second primaries may allow curative treatment and improve outcomes in this vulnerable population.

## Conclusions

To our knowledge, this is the first documented case of a second primary subglottic SCC following CHOP chemotherapy for non-Hodgkin lymphoma in a patient without conventional head and neck cancer risk factors. This rare occurrence underscores the need for long-term surveillance and prompt evaluation of new symptoms in lymphoma survivors.

Our report highlights the importance of considering therapy-related secondary malignancies in patients who present with atypical head and neck complaints after remission from lymphoma. Early recognition, appropriate imaging, and timely referral are crucial to improve outcomes in this rare clinical setting. Further studies are needed to better understand the potential carcinogenic effects of systemic chemotherapy and the mechanisms underlying second primary tumour development in this patient population.
